# Supplementary figures and images for: Expression of Spred2 in the urothelial tumorigenesis of the urinary bladder
Source: PLoS One. 2021 Nov 24;16(11):e0254289. doi: 10.1371/journal.pone.0254289 (PMC8612556; doi:10.1371/journal.pone.0254289)

Fig S1

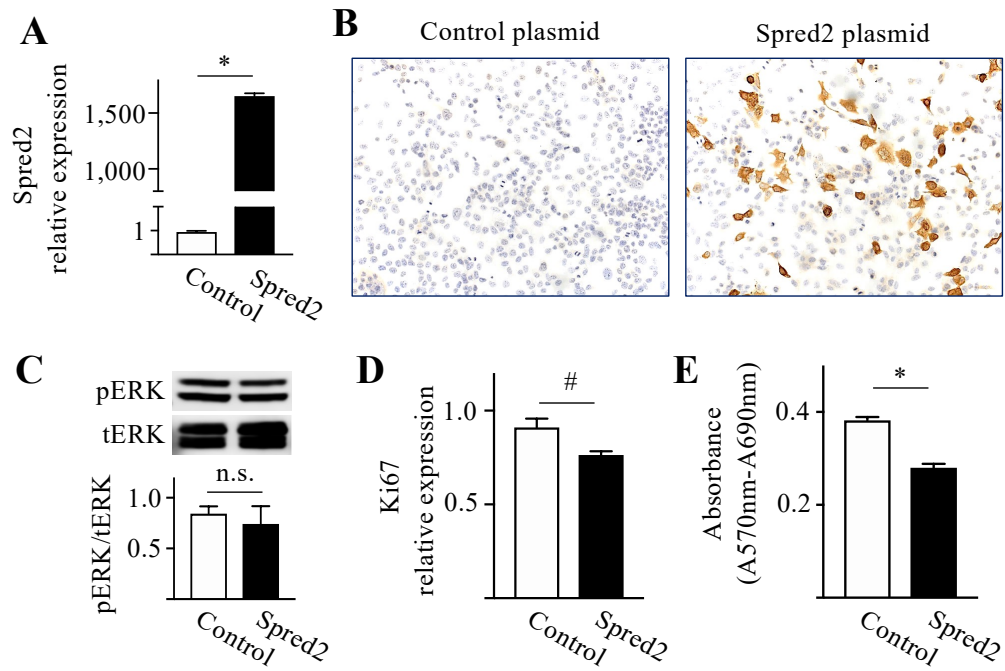

Supplement: S1 Fig — H1993 cells were transfected with Spred2 expression plasmid (OriGene, Rockville, MD, USA) or control plasmid (OriGene) using turbofectin 8.0 (OriGene). (A) Spred2 mRNA expressions after transfection with control plasmid (control) or Spred2 expression plasmid (Spred2) were analyzed by RT-qPCR (n = 3 each). Data is presented as mean ± SEM. *p<0.0001 (unpaired t test). (B) Transfection was carried out on Lab-Tek II Slide (8 Chamber, Electron Microscopy Sciences, Hatfield, PA, USA). The cells were fixed in 95% ethanol and immunostained with anti-Spred2 polyclonal antibody using the polymer method (Polink-2 Plus HRP RABBIT with DAB kit, GBI, Bothell, WA, USA). Spred2 positive cells were shown in brown. (C) Cell extracts after transfection with control plasmid (control) or Spred2 expression plasmid (Spred2) were immunoblotted with indicated primary antibodies (n = 3 each). Upper: Representative photos were shown. Lower: Band densities were digitised and semi-quantitated. Data is presented as mean ± SEM. (D) Ki67 mRNA expressions after transfection with control plasmid (control) or Spred2 expression plasmid (Spred2) were analyzed by RT-qPCR (n = 3 each). Data is presented as mean ± SEM. #p<0.05 (unpaired t test). (E) After transfection with control plasmid (control) or Spred2 expression plasmid (Spred2), the cell proliferation was evaluated by CCK-8 assay (n = 3 each). Data is presented as mean ± SE. *p<0.0001 (unpaired t test). (PDF) [file pone.0254289.s001.pdf]

Fig S2

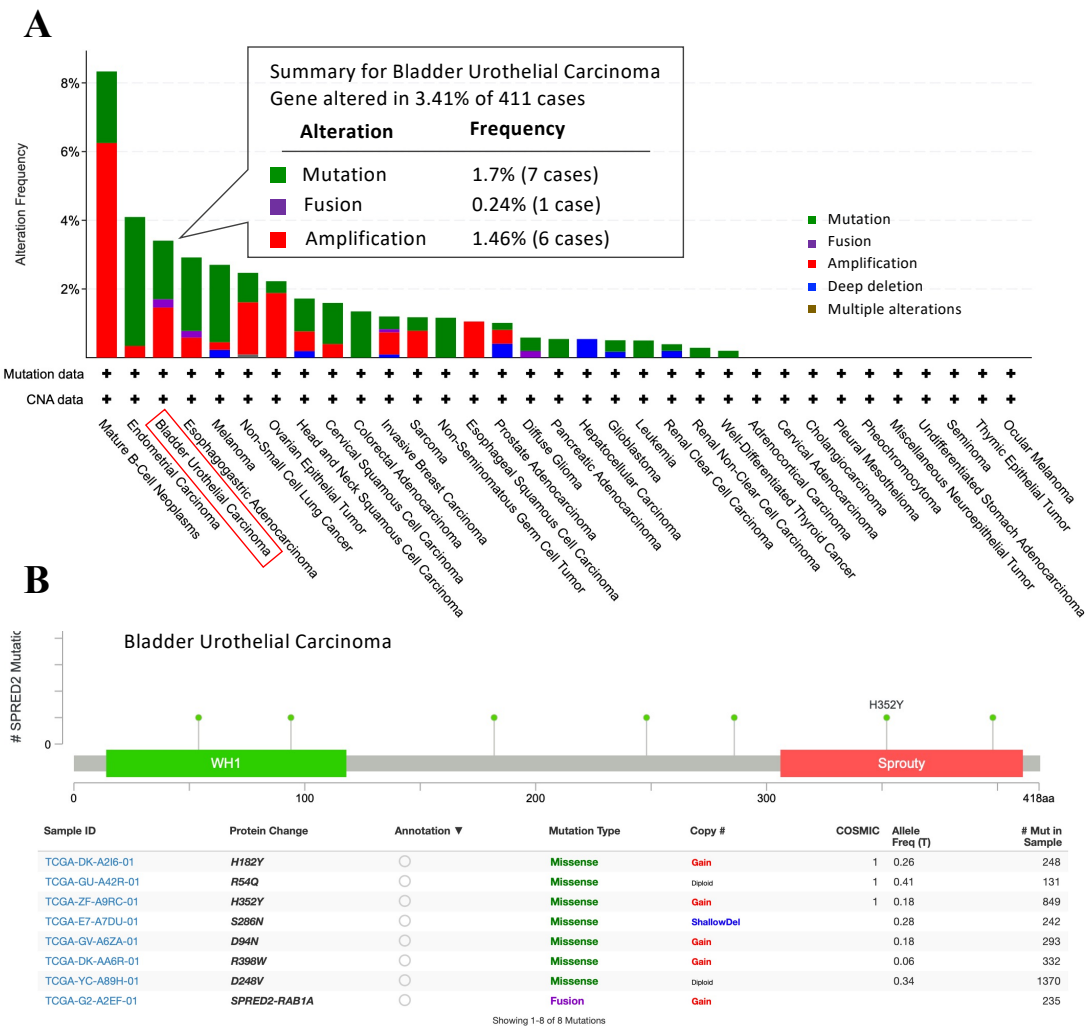

Supplement: S2 Fig — Data were from TCGA Pancancer Atlas from cBioPortal for Cancer Genomics (https://www.cbioportal.org/results/plots). (A) The Spred2 mutations in different cancer. Bladder cancer is the 3rd place having mutation of Spred2 among cancers. (B) The distribution of mutations on the domain structure of Spred2 in bladder urothelial carcinoma. (PDF) [file pone.0254289.s002.pdf]
